# Vegan milk and egg alternatives commercialized in Brazil: A study of the nutritional composition and main ingredients

**DOI:** 10.3389/fpubh.2022.964734

**Published:** 2022-10-31

**Authors:** Bernardo Romão, Raquel Braz Assunção Botelho, Eduardo Yoshio Nakano, Vinícius Ruela Pereira Borges, Maria Eduarda Machado de Holanda, António Raposo, Heesup Han, Miseldra Gil-Marín, Antonio Ariza-Montes, Renata Puppin Zandonadi

**Affiliations:** ^1^Department of Nutrition, University of Brasília, Brasília, Brazil; ^2^Department of Statistics, University of Brasília, Brasília, Brazil; ^3^Department of Computer Science, University of Brasília, Brasília, Brazil; ^4^CBIOS (Research Center for Biosciences and Health Technologies), Universidade Lusófona de Humanidades e Tecnologias, Lisboa, Portugal; ^5^College of Hospitality and Tourism Management, Sejong University, Seoul, South Korea; ^6^Public Policy Observatory, Universidad Autónoma de Chile, Santiago, Chile; ^7^Social Matters Research Group, Universidad Loyola Andalucía, Córdoba, Spain

**Keywords:** vegan products, dairy alternatives, egg substitutes, plant-based protein, dairy-free cheese

## Abstract

Worldwide, there is an increasing demand for plant-based food due to sustainable, health, ethical, religious, philosophical, and economic reasons. In Brazil, 14% of the population declares themselves vegetarians, and a noticeable increase in the consumption of vegan products is also noted. Substitution of animal dairy and egg is challenging from both sensory and nutritional aspects. Yet, there are no data regarding the nutritional value and ingredients of Brazilian commercial dairy and egg substitutes. Therefore, this study aimed to analyze the nutritional composition and used ingredients in Brazilian commercial vegan alternatives to dairy and eggs to provide information for Brazilian consumers of these products. A cross-sectional quantitative study was carried out in three steps: (i) sample mapping. (ii) data collection, and (iii) statistical analysis. A total of 152 samples were included. No differences were found between the energy value and total fat of vegan products and their animal counterparts. Vegan products showed higher amounts of carbohydrates and dietary fiber, and only the vegan versions of beverages and cheeses showed less protein than their counterparts. Cashews, rice, coconut, and soy were the most used ingredients in dairy substitutes. Emulsions of oil, starch, and isolated protein were used in vegan egg products. Most vegan beverages presented sugar in their composition. Vegan alternatives of dairy and eggs might be suitable for substituting their animal counterparts, but given that traditional versions of cheeses and milk are sources of protein in omnivorous diets, for equivalent nutritional replacement in vegan products, it is necessary to improve the protein content of their vegan counterparts.

## Introduction

Worldwide, there is increasing demand for plant-based food as an increasing number of people adopt vegan or vegetarian diets for sustainable, health, ethical, religious, philosophical, and/or economic reasons (1, 2). Vegetarianism is spreading across continents, with prevalences of 19% in Asia, 16% in Africa, 8% in 50 South and Central America, 6% in North America, and only 5% in Europe (3). In Brazil, 14% of the population identifies as vegetarians (4). Plant-based foods have been commercialized and marketed to meet the demand of vegans and vegetarians, as well as by individuals who suffer from lactose intolerance or are allergic to cow's milk or eggs (5, 6). Substitution of dairy and egg products is challenging from both sensory and nutritional aspects, raising questions about the nutritional value and ingredients of Brazilian commercial dairy and egg substitutes.

Cow's milk is a common staple food, and its consumption is present in the dietary habit of almost 6 billion people (7, 8). In countries such as India, the European Union—EU), and the United States—US), its yearly consumption revolves between 83 to 21 million metric tons (7). In addition to its consumption in the unprocessed form, milk derivatives such as yogurts and cheeses are highly present in the dietary habit of the overall world population (9). Chicken eggs are another staple food in the world's food habit, with its consumption being an average of 161 eggs per capita/year (10). Both foods stand out for their nutritional quality as they are sources of high biological value proteins, saturated fats, vitamins, and minerals (11, 12). Yet, milk and eggs and their derivatives contribute to desirable technological and sensory characteristics in various food preparations (13). Therefore, finding alternatives to milk and eggs in food is challenging, and the food industry is constantly searching for plant-based alternatives.

The use of plant-based milk alternatives has already been implemented since the end of the 20th century, and currently, it constitutes a market valued at around 3.9 billion dollars (USD) (14). As for the egg substitutes, although there is no data regarding the market value for this specific subject, projections regarding the plant-based alternatives market demonstrate continuous growth, especially in the Americas and Europe (15, 16). Consumers often look for options that sensorily mimics the characteristics of cow's milk and eggs. However, since these characteristics depend on intrinsic compounds of animal origin, there may be divergences in their plant-based counterparts' nutritional value and ingredient list (17–19). Commonly, plant-based milk alternatives consist of water-soluble extracts based on soybeans, cashews, almonds, coconut, hazelnuts, pseudocereals, and legumes (17). For egg substitutes, different ingredients are used according to the product's characteristics; usually, it consists of emulsions of vegetable fats or isolated vegetable proteins (20–22).

Multiple studies have described the nutritional composition of plant-based cow's milk alternatives, mainly from versions developed by researchers (12, 23–34). In general, lower protein, energy, and calcium concentrations were noted, with beneficial bioactive compounds such as β-sitosterol and β-glucan (17, 32). However, few studies have analyzed the nutritional composition and ingredients of both vegan milk derivatives and vegetable alternatives for egg-based products, especially regarding versions marketed and available to the population (9, 35–37). Therefore, there is a knowledge gap in this data. To our best knowledge, there are no studies conducted in Brazil on the nutritional composition and ingredients used in commercial vegan substitutes for dairy and eggs. So this study aimed to analyze the nutritional value and ingredients as described in data offered by food labels to provide information for consumers of vegan alternatives for dairy and eggs.

## Materials and methods

A quantitative, comparative cross-sectional study was performed in three steps: (i) sample mapping. (ii) data collection and classification, and (iii) statistical analysis.

### Sample mapping

The inclusion criteria for the milk and egg substitute samples in the study were (i) products sold in market chains with coverage in Brazil's five regions and/or food stores with national or regional coverage and (ii) products with the “Vegan Product” seal, provided by the Brazilian Vegetarian Society (SVB^®^). Fresh food and vegan products that do not intend to mimic animal-based milk or eggs were not included. The Brazilian law classifies as “Light” a product with a 25% reduction of a component; and as “Diet,” a product with total exclusion of a component (38). In this manner, products with nutritional claims of “Light” and “Diet” on salt, sodium, sugar, or total fat were excluded to avoid possible bias in the results (38). E-commerce was consulted through search platforms (Google^®^ Bing^®^), Brazilian online vegan products resellers, and on social media (Instagram^®^, Facebook^®^, and Twitter^®^) through hashtags and nominal searches to achieve national coverage of the milk and egg substitutes sold in the Brazilian market. The investigation was conducted from February 1st, 2021, to January 1st, 2022. The search was conducted in 4 stages, following a previously established protocol (39): (i) first, a researcher searched for vegan products; (ii) then a second researcher repeated the search process and analyzed if there were any missing products according to the established criteria; (iii) as a result, two independent academics double-checked the precision of the extracted data; and (iv) finally, a third coordinating researcher critically analyzed the data, determining the final group of samples. After the search, the included milk alternatives were classified as beverages, yogurts, and cheese, and the egg alternatives as mayonnaise and eggs. For comparison purposes, manufacturers of the best-selling animal products in Brazil were mapped, according to the Brazilian Association of Animal Proteins (BAAP^®^). Subsequently, three samples corresponding to each category of vegan products were included for further comparison of the nutritional composition.

### Data collection

Data collection was performed according to previous studies (9, 36, 37, 39, 40). The qualitative and quantitative data reported on the products' food labels were recorded, including firm name, brand name, descriptive name, ingredient list, nutrient information, and serving size. Information about the ingredients and nutrient values was collected from the food label. According to the Brazilian legislation, it is mandatory to describe the serving size (g), energy value (kcal), carbohydrates (g), added sugars (g), proteins (g), fats (g), saturated fats (g), dietary fiber (g) and sodium (mg) (38). Nutrients with an optional declaration, such as added sugar (g) were also collected when available. The nutritional value of powdered versions of beverages (e.g., Powdered soy milk) was included proportionally as the concentration of nutrients according to their label suggested dilution. For standardiation and comparison purposes, all values were converted to a serving size of 100 g. According to Brazilian legislation, nutritional labels can be based on food composition tables and present a discrepancy level of 20% (for more or less) between its actual chemical composition and that described on the label (38). Thus, possible divergences may be present, as evidenced by other studies utilizing food labels as their information source (9, 19, 37, 39, 40).

### Statistical analysis

Data regarding the included samples' energy value (kcal), carbohydrates (g), added sugars (g), proteins (g), fats (g), saturated fats (g), dietary fiber (g), and sodium (mg) were calculated on their respective means ± standard deviations (SD). A comparison between nutritional values of milk and egg substitutes and their respective animal protein-based products was performed using a non-parametric Mann-Whitney' test with a confidence level of 95% (*p* < 0.05). Two-tailed hypotheses were considered in the test. Microsoft Excel^®^ (USA, 2021) and SPSS^®^ version 22.0 (IBM SPSS Statistics, Version 22.0, IBM corp., Chicago, IL USA, 2020) were used to perform the tests.

For graphical visualization, a word cloud was generated with the utilized ingredients of vegan milk and egg analogs, given that higher frequencies are represented with more prominent words in the cloud (Wordclouds^®^, 2022) (41). For word cloud generation, protein sources were grouped according to their main matrix; for example, coconut cream, shredded coconut, and dry coconut were all classified as “coconut.” The range of the nutritional values was expressed visually through the parallel coordinate's technique, where the minimum and maximum values are depicted at the bottom and the top of the axis, respectively (42). Furthermore, information regarding the ingredients was represented by percentages in a heatmap where the color indicates the ingredient's presence according to the stipulated categories. GraphPad Prism^®^, (San Diego, CA, USA, 2022) was used to generate the heatmaps.

## Results

A total of 152 samples were included in the study. From all samples, 89.47% were vegan alternatives of milk derivatives (*n* = 136), given that from all samples, 52% were classified as beverages (*n* = 80), 7.2% as yogurts (*n* = 11), and 29.6% as cheese (*n* = 45). 10.52% of the samples were classified as egg replacers (*n* = 16), given that from all included products, 9.2% were classified as mayonnaise (*n* = 14) and 1.3% as eggs (*n* = 2). Table 1 presents the vegan and animal samples' energy value (kcal), carbohydrates (g), proteins (g), fats (g), saturated fats (g), dietary fiber (g), and sodium (mg) by means and standard deviations (SD). Complete information regarding nutritional value, ingredient list, and serving size in all included samples is available in the Supplementary Table S1.

**Table 1:** Means and standard deviations (SD) of the nutritional values per 100 g of serving of the included samples.

**Samples**	**Energy (Kcal)**			**Carbohydrates (g)**			**Protein (g)**			**Total Fat (g)**			**Saturated Fat (g)**			**Dietary Fiber (g)**			**Sodium (mg)**		
	**Vegan**	**Animal**	**p**	**Vegan**	**Animal**	**p**	**Vegan**	**Animal**	**p**	**Vegan**	**Animal**	**p**	**Vegan**	**Animal**	**p**	**Vegan**	**Animal**	**p**	**Vegan**	**Animal**	**p**
Beverages	40.80 ± 14.36	46.00 ± 11.95	0.602	5.31 ± 3.77	4.63 ± 0.32	0.918	**1.05** **± 0.87**	**3.10** **± 0.10**	**0.002**	1.69 ± 1.27	1.50 ± 1.50	0.918	0.37 ± 0.50	1.05 ± 1.00	0.304	0.63 ± 0.95	0 ± 0	0.098	**30.65** **± 22.88**	**69.33** **± 1.53**	**0.05**
Yogurts	78.52 ± 37.98	70.31 ± 32.94	1,000	7.56 ± 4.92	8.51 ± 5.88	0.368	1.70 ± 2.21	3.74 ± 0.23	0.225	4.67 ± 5.16	2.31 ± 2.11	0.769	3.31 ± 1.96	1.41 ± 1.31	0.06	**0.69** **± 0.49**	**0** **± 0**	**0.011**	**37.43** **± 39.87**	**85.66** **± 52.13**	**0.032**
Cheese	281.89 ± 87.24	265.56 ± 5.09	0.657	**13.97** **± 8.23**	**2.89** **± 0.77**	**0.001**	**5.11** **± 4.24**	**9.89** **± 1.84**	**0.032**	27.85 ± 33.97	23.89 ± 0.19	0.873	6.81 ± 6.28	14.78 ± 0.96	0.066	0.95 ± 1.15	0 ± 0	0.156	348.43 ± 268.48	445.56 ± 56.80	0.213
Mayonnaise	320.36 ± 145.56	391.67 ± 250.97	0.591	4.62 ± 3.98	8.33 ± 0.83	0.068	2.77 ± 5.43	0 ± 0	0.197	31.68 ± 18.63	40.28 ± 29.37	0.509	**4.27** **± 3.75**	**21.67** **± 25.04**	**0.012**	55.95 ± 209.35	0 ± 0	0.859	597.74 ± 221.7	830.56 ± 258.92	0.244
Eggs	325.00 ± 106.07	557.48 ± 45.15	0.2	**25.07** **± 11.21**	**5.61** **± 3.42**	**0.02**	42.64 ± 2.32	45.27 ± 5.02	0.8	12.57 ± 6.46	38.62 ± 3.96	0.2	**0** **± 0**	**12.6** **± 1.64**	**0.02**	**13.43** **± 0.81**	**0** **± 0**	**0.02**	240.00 ± 339.41	507.52 ± 25.09	0.4

It is considered statistically different when p < 0.05, at Mann-whitney's test, with results highlighted in bold numbers.

No differences between vegan and animal products regarding energy values (kcal) were found. Cheese and eggs vegan options presented higher carbohydrates than their animal counterparts. The vegan cheese alternatives presented an average of 389% more carbohydrates than their counterparts; vegan eggs presented 339% more carbohydrates than their animal counterparts.

Animal products presented higher protein concentrations than vegan alternatives only in the categories of beverages and cheese. A difference of 61% in protein content was found between vegan and animal beverage samples, while in the cheese category, the difference was 93%.

No differences were found in total fat between vegan and animal samples. Greater concentrations of saturated fats were found in the animal counterparts of mayonnaise (21.67 ± 25.04 g/100 g) and eggs (12.6 ± 1.64/100 g); statistical differences were found only in these groups.

Yogurt and egg vegan alternatives presented higher dietary fiber than their animal counterparts (Table 1). Animal-based beverages and yogurts presented higher amounts of sodium than their vegan counterparts. There were no significant differences between other animal and vegan products.

**Figure 1:**
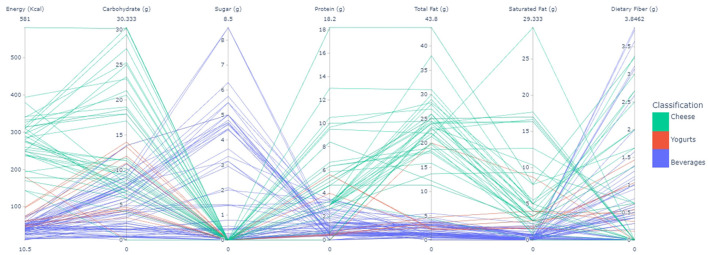
Range of the nutritional values of the included milk derivatives.

**Figure 2:**
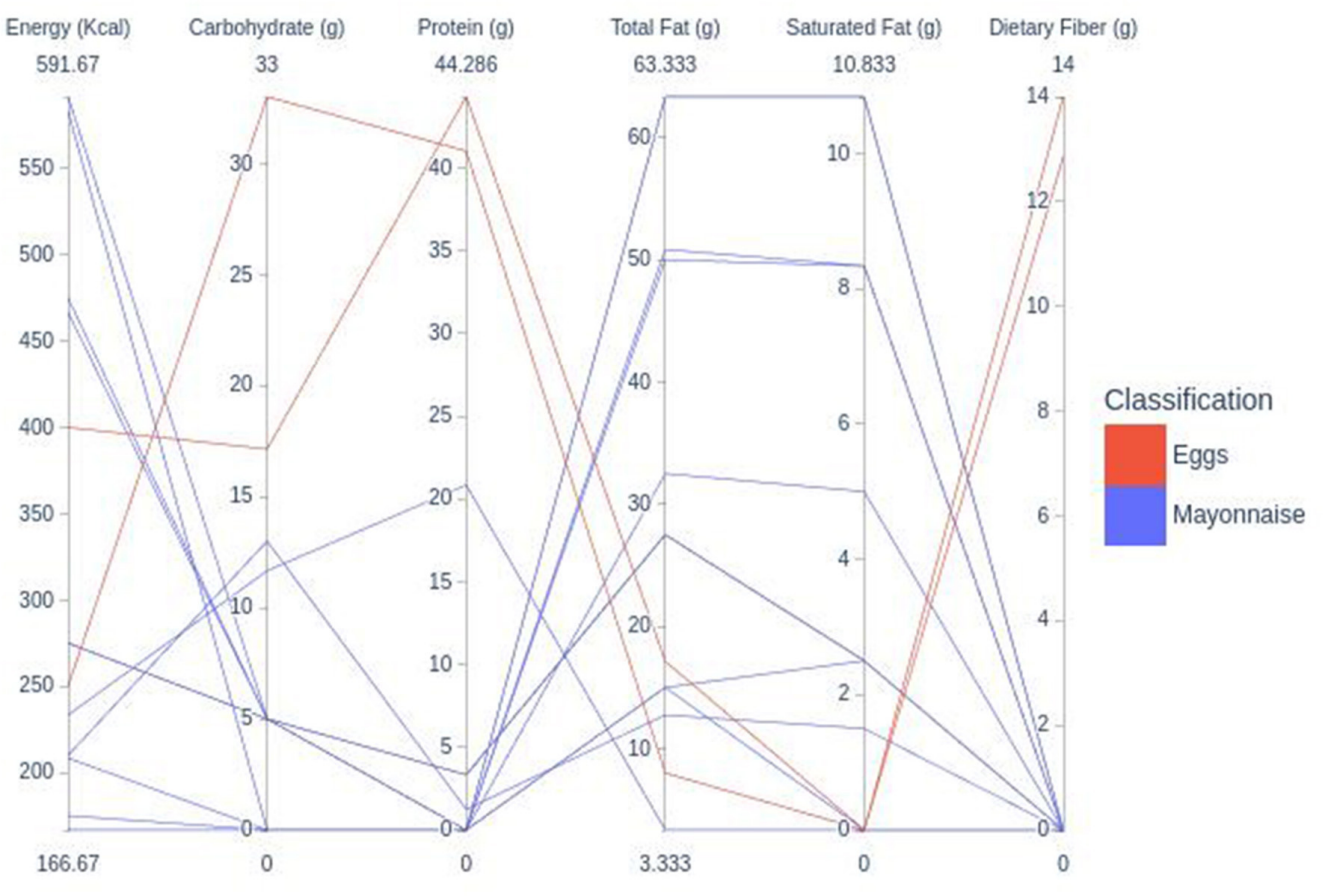
Range of the nutritional values of the included egg substitutes.

A comparison between the range of the nutritional values of the evaluated samples (standardized per 100 g of product, maximum and minimum values) according to their categories is presented in Figure 1 for beverages, yogurts, and cheese and Figure 2 for mayonnaise and eggs. Figure 1 highlights that most vegan beverages tend to present energy values lower than 100 kcal/100 g: up to 14% of carbohydrates, up to 4% of protein, up to 5.2% of total fat, up to 2.5% of saturated fat, and up to 4% of dietary fiber. The sugar content varied greatly in samples, from 0 to 8.5%. In yogurts, the samples' energy values ranged from 70-100 kcal/100 g: up to 7% of carbohydrates, 2% of protein, 5% of total fat, 2% of saturated fat, and 0.7% of dietary fiber. As for cheese, the included samples' energy values ranged from 180–300 kcal/100 g, presenting up to 14% carbohydrates, 5% protein, 28% total fat, 7% saturated fat, and 1% of dietary fiber.

In Figure 2, mayonnaise presented energy values ranging from 186 to591 kcal/100 g, presenting carbohydrates, protein, total fat, saturated and dietary fiber of 13, 2, 30, 4, and 13%.

**Figure 3:**
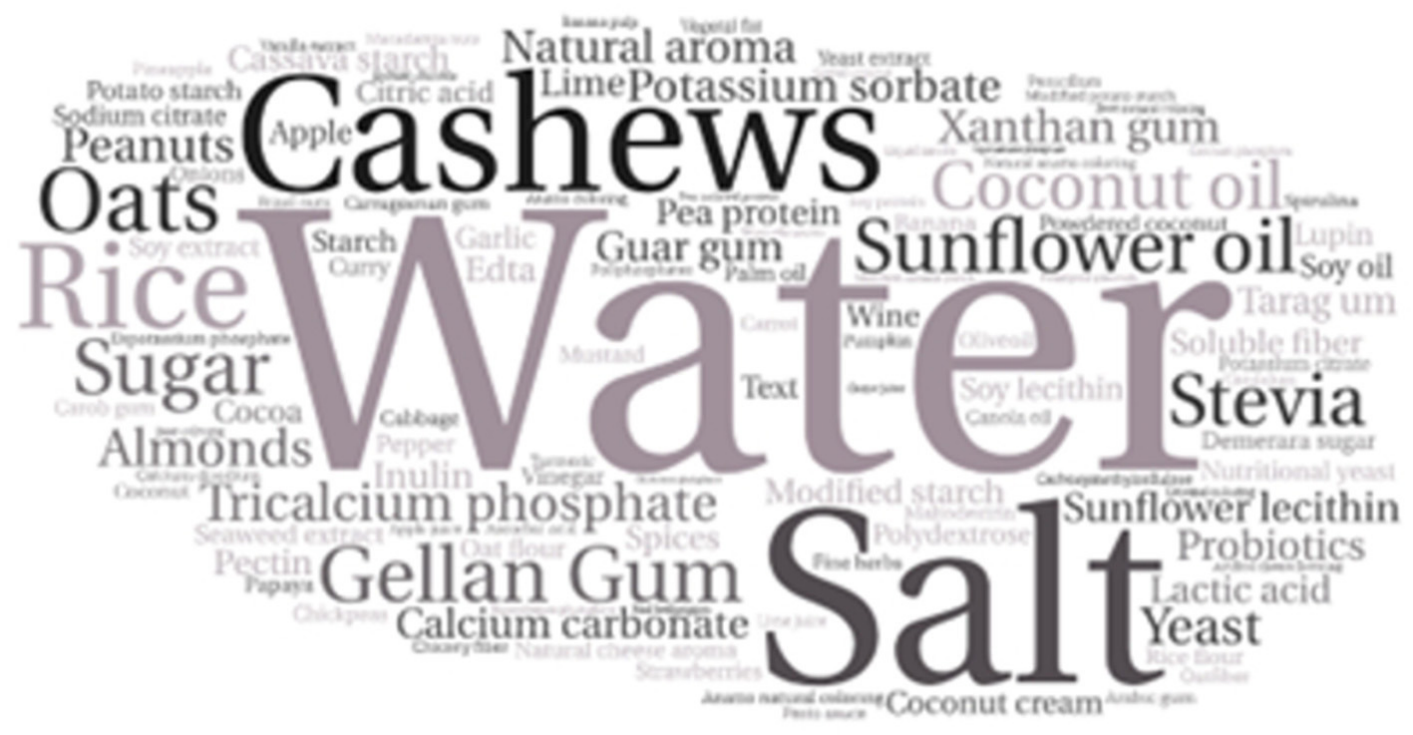
Word cloud generated from the frequencies of used ingredients in beverages, yogurts, and cheese.

**Figure 4:**
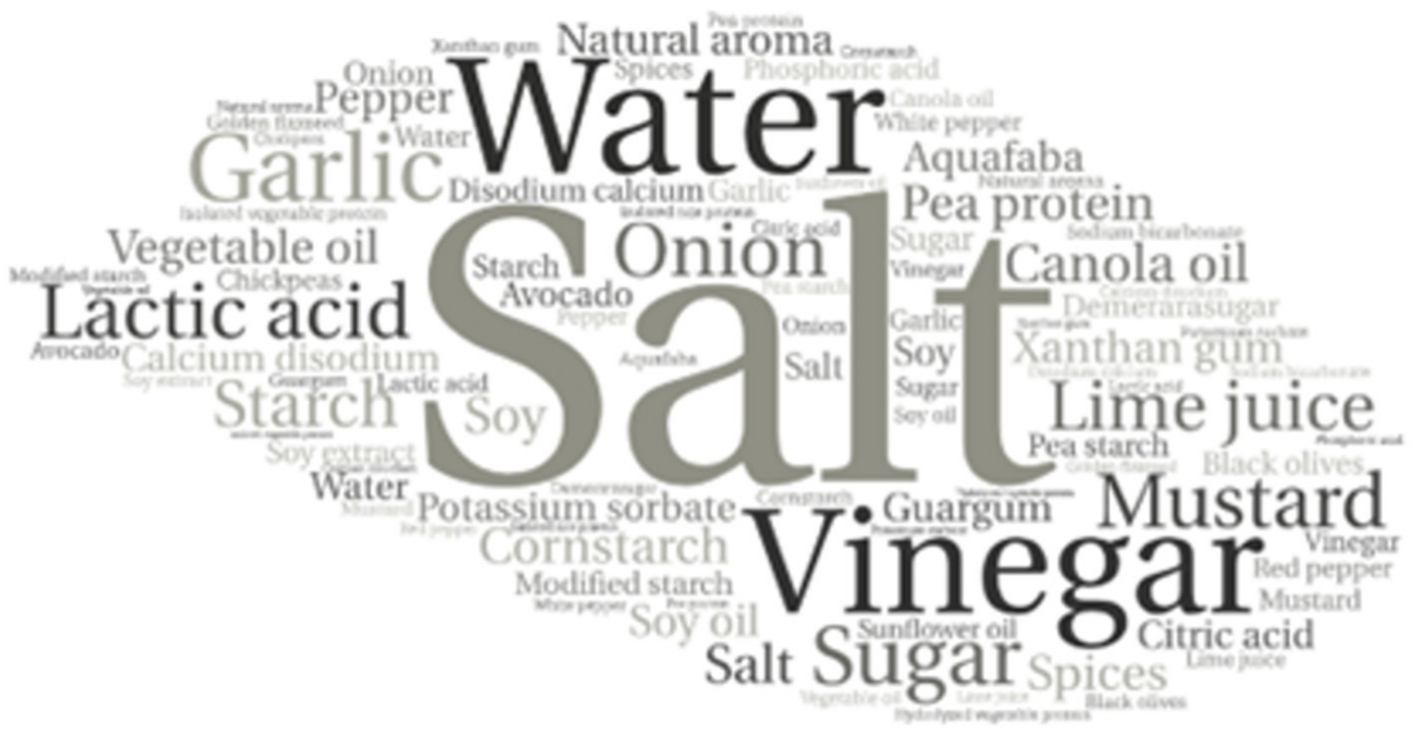
Word cloud generated from the frequencies of used ingredients in mayonnaise and eggs.

The word clouds generated from the frequencies of the ingredients of the included samples is expressed in Figure 3 for the milk derivatives (beverages, yogurts, and cheese) and Figure 4 for egg replacers, mayonnaise, and eggs.

Among the vegan milk derivatives (89.4% of the samples), the most used ingredient was water, present in 81.6% of the samples, followed by salt, present in 68%. Sugar was present in 60% of the samples. Regarding the main matrixes utilized in these products, cashews were the most used (38.2% of the samples), followed by coconut (22%), rice (16.9%), oats (14.7%), soy (13.9%), almonds (13.2%), pea protein (11.7%), peanuts (5.14%), and carob (2.9%). Combinations of two or more sources functioning as main matrixes were found in 17% of the samples, being the combination of rice and coconut, the most frequent one (56%), coconut and cashews (26%), oats and cashews (14%), and soy and pea protein (11%). Coconut oil was the most used fat source (25% of the samples), followed by soy oil, only 2.9%. Food additives such as natural aroma [volatile molecules with aromatic properties extracted from natural products (38)], calcium carbonate, tricalcium phosphate, sodium citrate, and potassium citrate were used in 49.2, 35, 22.7, 3.6, and 3.6%, respectively. Also, thickeners and gums were used, in the form of gellan gum (26.4%), xanthan gum (18.3%), modified starch (13.9%), polydextrose (5.1%), tara gum (4.4%), inulin (2.9%), carob gum (2.9%) and Arabic gum (2.9%).

Regarding the egg replacers, salt and vinegar were present in 100% of the samples, followed by lactic acid (85%), starch (72%), lime juice (71%), mustard (71.4%), garlic (71.4%), sugar (64.2%), potassium sorbate (64.2%), calcium disodium (64.2%), unspecified vegetable oil (42.8%), natural aroma (42.8%), canola oil (35.7%) and pea protein (35.7%).

**Figure 5:**
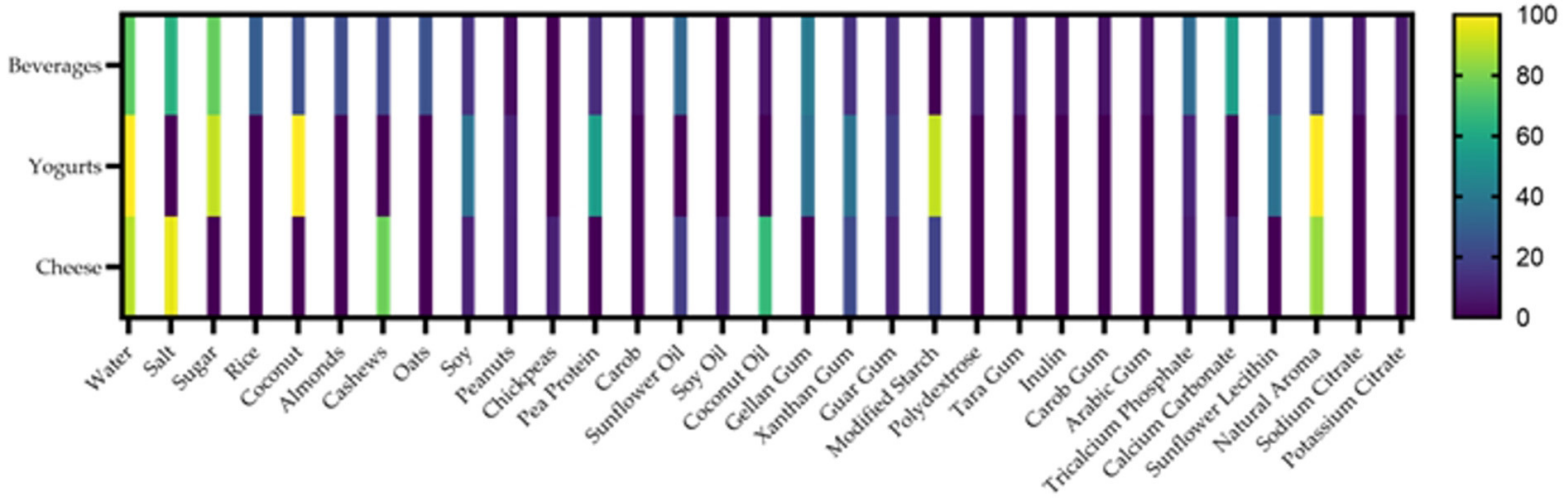
Percentages of frequencies of implemented ingredients in vegan milk derivatives in a heatmap. Colored clusters represent different frequencies according to the scale placed on the right side.

The percentual distribution of the frequency of ingredients in the group of milk derivatives with frequencies higher than 1.5% among specified categories is described in the heatmap in Figure 5. The heatmap of egg and mayonnaise was not constructed due to the low number of samples in the market.

Water was the most common ingredient in 76.3% of the beverages, followed by sugar in 75%% of the products. Salt was present in 63% of the samples. As for the main matrixes, rice was the most abundant one, utilized in 28.7% of the samples, while other matrixes such as oats, coconut, almonds, cashews, soy, peanuts, pea protein, and carob were utilized in 25, 23.7, 22.5, 13.75, 2.5, 12.5, and 5% respectively. Sunflower oil was the most used fat source, present in 32% of the samples. Coconut oil was utilized in 5% of the samples. Thickeners were also included in this category, such as gellan gum (40%), xanthan gum (13.7%), guar gum (12.5%), polydextrose (8.7%), tara gum (7.5%), inulin (5%), carob bum (5) and Arabic gum (5%). Calcium carbonate was the most utilized food additive in 55% of the samples. Other food additives, such as tricalcium phosphate, sunflower lecithin, natural aroma, sodium citrate, and potassium citrate, were used in 33.7, 22.5, 22., 6.25, and 6.25%, respectively.

Regarding yogurts, water was used in 100% of the samples. Sugar was present in 90.9% of the included products. Coconut and pea proteins were the most common matrixes in this category, given that coconut was utilized in 100% of the samples and pea protein in 54.4%. Other matrixes, such as soy (36.3%) and peanuts (9%) were also used. Modified starch was used in 90% of the samples, followed by xanthan and gellan gum (36.3%) and sunflower lecithin (36.3%). The natural aroma was present in all samples (100%).

Among cheese samples, salt was present in 96% of the samples, and water in 88.8%. Cashews were the main matrix in this category, present in 77.7% of the samples. Other used matrixes were soy (8.8%), peanuts (8.8%), and chickpeas (8.8%). Modified starch was also utilized as a matrix in 20% of the samples. Coconut oil was the primary fat source, used in 66.6% of the samples, followed by sunflower oil, 17.7%, and soy oil 8.8% Xanthan gum was present in 22.2% of the samples, and guar gum at 8.8%. Food additives such as natural aroma (84.4%), calcium carbonate (8.8%), and tricalcium phosphate (6.6%) were also used in these samples.

In the mayonnaise category, lactic acid was present in 100% of the samples, starch in 85%, lime juice in 85%, mustard in 71%, garlic in 71%, sugar 64%, potassium sorbate 64%, calcium disodium 64%, unspecified vegetable oil in 56% and canola oil in 50%.

The main ingredients in the vegan egg category were isolated proteins (pea protein, 100%) and starch and chickpea flour (100% of the samples).

## Discussion

Plant-based vegan diets are strongly associated with better health status, given their bioactive compound-rich nature, and usually provide less calories, equal protein, and more dietary fiber (43–46). However, vegan industrial products aiming to replace their animal counterparts tend entail disadvantages, with potential nutritional impairments (39).

Regarding the energy values of the studied samples, no differences were found between vegan products and their animal counterparts. The higher amount of carbohydrates in the vegan alternatives probably balanced the energy value to a level of similarity between animal and vegan versions. Usually, vegan products tend to present lower energy values, given that plant-based matrixes are commonly poor in compounds with a higher energy density, such as saturated fats (19, 36, 39, 40). This tendency is also evident in vegan milk and egg derivatives, as found by other studies analyzing developed formulations and different markets worldwide (17, 26, 28, 36, 37).

Vegetables are known sources of carbohydrates; however, differences were found only between animal and vegan versions of cheese and eggs, given that these products are based on nuts and legumes groups of foods naturally rich in carbohydrates (47, 48). Nevertheless, being carbohydrate-rich is a common feature among all plant-based alternatives, regardless of their purpose of substitution (milk, eggs, meat, poultry, etc.) (17, 19, 39, 40, 49, 50). Also this study's results suggest an evident pattern regarding the proportion of this nutrient in plant-based milk and egg alternatives around the world (17, 34, 37, 40, 51).

In their animal versions, both milk derivatives and eggs are well known for providing high-quality protein (casein, whey protein, and albumin) (11, 52), usually presenting higher concentrations of this nutrient in comparison with their vegan counterparts (17, 22). In our study, animal counterparts presented higher concentrations of protein. However, differences were found only regarding beverages and cheese. Plant-based beverages are commonly made of water-soluble extracts of cereals, pseudocereals, and general pulses such as soy, oats, coconut, cashews, and almonds (23), and this type of ingredient tends to present lower concentrations of protein, especially when in comparison to dairy (17). Another issue regarding plant-based beverages revolves around protein quality, specifically concerning the present amino acids in their formulations. Vegetable proteins usually present limitations regarding the amino acids in their structure, with lower levels of essential amino acids, more specifically, methionine and lysine (53, 54). Thus, to better assimilate vegetal protein, two or more sources should be combined (54). However, it is important to note that the present study did not determine the protein bioavailability or its amino acid composition. Therefore, future studies regarding this specific subject should be performed.

A similar pattern regarding plant-based beverages worldwide was found in the included samples. Usually, commercial alternatives are frequently based on soy, cashews, coconut, and almonds, respectively, with versions providing combinations of two or more sources for better nutritional and sensory aspects (24, 26, 40, 51). However, in our study, different from those in other countries, the most frequent matrix was rice instead of soy (51, 55), followed by oats, coconut, and almonds. The controversies around soy consumption and its effect on consumers' perception of health issues probably led to a decrease in the production of soy-based beverages. Also, vegetal beverages are widely consumed by infants; therefore, increasing the prevalence of soy allergy in infants may decrease the use of soy as a matrix for these products (56, 57). Given the protein-rich nature of soy, its reduced use might have exerted an effect on our findings regarding the lower protein quantity in vegan beverages (1.05 ± 0.87/100 g) than in commercial cow's milk (3.10 ± 0.10 g/100 g) (58). As for the included vegan yogurts, coconut was the most utilized matrix, probably because its higher fat content may result in a product with improved viscosity, texture, lubricity, and taste, especially in comparison with animal-based yogurt, a product known for these characteristics (59, 60). Yet, although coconut was used as the main ingredient, soy and pea protein were also frequently incorporated, contributing to higher protein concentrations with no differences from their animal counterparts.

Another main concern regarding plant-based beverages is the presence of sugar. In cow's milk, a striking sensory characteristic is a mild sweetness provided by lactose (52). In an attempt to mimic this sensory characteristic, most plant beverages implement sucrose replacing lactose (25). One point to be considered is that although both molecules are disaccharides, because of their molecular nature, sucrose has a considerably stronger sweet power than lactose (61). In addition, excessive sucrose consumption is a public health problem, as it may increase the prevalence of chronic non-communicable diseases (47).

Currently, the worldwide recommendation for sucrose consumption is 50 g/day (47). Therefore, considering that according to our study, a usual serving of 200 g of vegetable beverage presents on average 16 g of sucrose, a serving of vegetable beverage results in 32% of the recommended daily sugar intake in only one portion.

Animal-based cheese is usually produced from the curds of coagulated milk (mostly β-casein) or even proteins derived from wastewater whey (13), with optional fermentation and different concentrations of fat (13). In the case of vegan cheese alternatives, cashews are primarily used for their saturated fat-rich nature, which provides better sensory characteristics, such as structure, stability, lubricity, and aftertaste (62). Yet, although better sensory aspects are obtained with cashews, nutritional impairments regarding protein content might be present. Similar to studies performed in other countries, Brazilian's vegan cheese alternatives are mainly based on cashews, with few samples being based on modified starch, soy, and peanuts (9, 36, 37). Also, the pattern of products with less protein than their animal counterparts and even quantities of total and saturated fat was also found (9, 36, 37). Cashews are part of the oilseeds category, seeds with a high concentration of different kinds of fats. In the case of cashews, these nuts are rich in saturated and monounsaturated fats, which give characteristics such as greater stability at room temperature, better texture, lubricity, creaminess, and aftertaste (13). Thus, this nut constitutes an adequate product to substitute characteristics provided by cow's milk fat in cheeses.

The vegan egg category did not show differences in protein content compared to their animal counterparts. Probably, since egg replacers were based on a mixture of isolated proteins (pea protein) and protein-rich flour (chickpea flour), this difference was not found. Nevertheless, different characteristics and technological applications broadly differentiate the two categories of products.

Animal eggs perform many roles in food products (13). Albumin, the main protein in chicken eggs, can stabilize bakery products, improving texture, mainly when used in foam form. Also, chicken egg's yolk is rich in phospholipids, natural emulsifiers that contribute to products' stability, durability, and texture, while providing desirable coloring given its carotenoid-rich nature (13). In this sense, although isolated proteins and legume flours are also used as ingredients that will enhance sensory characteristics similarly to animal eggs, their performance is reduced compared to animal eggs. Therefore, although there are no differences in the average value of proteins, the different molecular composition of these products results in different characteristics in the products in which they are used (63–65).

Generally, animal products present higher concentrations of total and saturated fat, and this tendency was also found in this study's results. More specifically, in egg substitutes (mayonnaise and eggs), higher amounts with significant differences in saturated fat were found, possibly, because chicken eggs used in this type of formulation present about 37% of saturated fat (11, 66). Vegetables are usually a source of mono and polyunsaturated fatty acids, with coconut and palm oil being the most prominent sources of saturated vegetal fats (22, 67, 68).

Coconut oil is a very interesting fat from an industrial point of view because it has resistance to high temperatures and is suitable for cooking. In addition, it can remain solid at room temperature, independent of hydrogenation processes (59, 69). More specifically, this oil was mostly used in this category of vegan cheese, probably because of its technological capacity to provide stability and texture while also improving its shelf-life. This can be due to lauric acid, an intrinsic fatty acid present in coconut oil, with fungicidal characteristics (59, 69).

In the case of vegan beverages, sunflower oil was the most used fat source. Sunflower oil is rich in monounsaturated fatty acids and widely implemented in industrial food processes because of its emulsifying capacity (70, 71). Plant-based beverages consist of non-homogenous solutions of water-soluble plant extracts (17). Therefore, the need for emulsifiers is highlighted, given that implementing sunflower oil provides a more stable, homogenous, and desirable sensory product (70, 71). Another sunflower-based product, sunflower lecithin, an efficient emulsifier, is also widely used among the included samples (beverages and yogurts), probably with the same objective (72). Mayonnaise consists of fatty emulsions, and vegetable oils and chicken eggs are commonly used (13). In the egg yolk, compounds of the phospholipid class stabilize the liquid and fatty phases resulting in a creamy and sensory-appropriate product (11). In the included vegan samples of mayonnaise, the same process is employed using unspecified vegetable oils and canola oil; however, in the absence of chicken eggs' phospholipids, starches are utilized to sustain the emulsion through rheological characteristics such as gelatinization (73).

To a large extent, vegan substitutes for meat and dairy tend to present higher amounts of dietary fiber (9, 35, 39, 40), given that vegetables are the best-known sources of this nutrient (74). In our study, although vegan versions presented generally higher amounts of fiber than their animal counterparts, differences were found only between vegan and animal versions of yogurts and eggs. This is probably because animal versions of both categories typically do not present any dietary fiber in their composition (66).

The consumption of dietary fibers is essential for maintaining health since it favors the intestine's functioning and normal blood glucose and cholesterol levels (75). Individuals who adhere to vegetarian and vegan diets usually consume adequate or higher amounts of fiber than those recommended by the dietary reference intakes (60, 61). However, the same is not true in Brazilian omnivore diets. Recent studies have shown that Brazilian eating habits present a decrease in the consumption of vegetables, whole grains, and legumes in favor of industrialized foods with lower amounts of these nutrients (76). Therefore, since there is no difference between vegan and animal yogurts regarding the protein value, the consumption of this combined with a balanced diet may contribute to improve this scenario.

Vegan options showed lower sodium concentrations, with significant differences between vegan and animal versions of beverages and yogurts, possibly due to the higher intrinsic sodium content of cow's milk and the use of preservatives whose composition also presents this compound (25, 52). Nevertheless, these results align with the trend presented by studies conducted in other countries where, in general, vegan versions presented lower sodium content than their animal counterparts (9, 35, 39, 40). Salt was utilized in most cheese vegan analogs, given that usually, cheese is a salty product.

One issue that emerges when comparing cow's milk analogs is the need for food additives. Food additives consist of legalized food substances, which are not nutrients, but add food-friendly technological characteristics (77). In the case of vegan milk derivatives, hydrocolloids such as xanthan, gellan, and carob gums were used in the samples. Hydrocolloids provide stability and texture to food preparations forming stable gels in water (78). In the case of beverages, yogurts, and mayonnaise, this characteristic contributes to the emulsion of the liquid (water) and solid (soluble vegetable extract) phases of the product (79, 80). In dairy-free cheeses and vegan mayonnaise, those gels are responsible for the final texture and stability at room temperature (9, 36).

Preservatives are another class of food additives that stands out in the included samples. Calcium carbonate, tricalcium phosphate, sodium citrate, and potassium citrate were widely used in yogurts, beverages, cheese, and mayonnaise. These preservatives contribute to the shelf-life extension of these foods by functioning as bactericidal substances and delaying the intrinsic enzymatic deterioration of these products (81). Moreover, calcium carbonate also acts as a calcium food supplement, enriching vegan preparations and making them comparable with their animal counterparts (17). However, according to Brazilian legislation (38) announcing the total calcium content on the products' food labels is not mandatory. Therefore, although many products present calcium carbonate in their composition, it was impossible to perform an accurate evaluation and comparison with animal counterparts.

One potential limitation of our study was the absence of laboratory chemical analysis to confirm the label information. As mentioned above, according to Brazilian legislation, nutritional labels can be based on food composition tables and present a discrepancy level of 20% (for more or less) between its actual chemical composition and that described on the label (38). Thus, possible divergences may be present, as evidenced by other studies utilizing food labels as their information source (9, 19, 37, 39, 40).

## Conclusions

From the above, it is possible to conclude that the market for vegan alternatives for dairy products and eggs is growing prosperously in Brazil. In general, no differences were found regarding the energy value of vegan and animal versions of all included samples. Only the categories of cheese and eggs presented more carbohydrates than their animal counterparts. Only the animal versions of cheese and beverages showed more protein than their vegan counterparts. No differences were found regarding the total fat content of the samples. However, the animal samples of mayonnaise and eggs showed greater saturated fats. Yogurts and vegan eggs showed more fiber than their animal counterparts. Regarding the ingredients, cashew nuts were the most used matrices in vegan dairy products, while rice and coconut were the most prominent in the included beverages. In vegan beverages, most of the samples presented added sugar. Gums, thickeners, and preservatives were the most widely used food additives. Finally, most samples of egg substitutes consist of mayonnaise-based on vegetable oil and starch emulsions with added isolated plant proteins. Total egg substitutes were based on proteins isolated in powder and legume flours. Vegan dairy and egg alternatives might be suitable for substituting their animal counterparts, but given that traditional versions of cheeses and milk are sources of protein in omnivorous diets, for equivalent nutritional replacement in vegan products, it is necessary to improve the protein content of their vegan counterparts.

## Data availability statement

The original contributions presented in the study are included in the article/Supplementary material, further inquiries can be directed to the corresponding authors.

## Author contributions

Conceptualization, methodology, investigation, data curation, and writing—original draft preparation: BR, RB, and RZ. Software: EN, MH, and VB. Validation: RB and RZ. Formal analysis: BR, RB, RZ, EN, MH, and VB. Resources: RZ. Writing—review and editing: BR, RB, AR, and RZ. Visualization: RB and AR. Supervision: AR, RZ, AA-M, and MG-M. Project administration: AR, RZ, and HH. Funding acquisition: AR and RZ. All authors have read and agreed to the published version of the manuscript.

## Conflict of interest

The authors declare that the research was conducted in the absence of any commercial or financial relationships that could be construed as a potential conflict of interest.

## Publisher's note

All claims expressed in this article are solely those of the authors and do not necessarily represent those of their affiliated organizations, or those of the publisher, the editors and the reviewers. Any product that may be evaluated in this article, or claim that may be made by its manufacturer, is not guaranteed or endorsed by the publisher.
