# Supplementary material for: Vegan milk and egg alternatives commercialized in Brazil: A study of the nutritional composition and main ingredients
Source: Front Public Health. 2022 Oct 31;10:964734. doi: 10.3389/fpubh.2022.964734 (PMC9667870; doi:10.3389/fpubh.2022.964734)
Supplement: Supplementary file 1 [file Table_1.DOCX]

Table S 1 - Information regarding serving size, ingredients and nutritional value of all included samples

| Category | Ingredients | Serving Size | Energy (kcal) | Carbohydrates (g) | Protein (g) | Total fat (g) | Saturated Fat (g) | Dietary Fiber (g) | Sodium (mg) |
| --- | --- | --- | --- | --- | --- | --- | --- | --- | --- |
| Beverages | water, pea protein, coconut oil, sugar, chicory fiber, pineapple, sunflower oil, natural aroma, salt, cabbage, vitamin d2, vitamin b12, dipotassium phosphate, calcium phosphate, arabic gum, gellan gum, EDTA. | 250 | 92 | 3.7 | 3.2 | 6.4 | 4.2 | 2.3 | 153 |
| Beverages | water, pea protein, sugar, chicory fiber, pineapple, coconut oil, sunflower oil, natural aroma, salt, cabbage, vitamin d2, vitamin b12, dipotassium phosphate, calcium phosphate, arabic gum, gellan gum, EDTA. | 250 | 60 | 3.4 | 3.2 | 3 | 2 | 9 | 152 |
| Beverages | water, almonds, coconut cream, pea protein, minerals (tricalcium phosphate), (guar gum, gellan gum, polyphosphates), sunflower lecithin, vitamins (B6, D2 and B12), natural aroma and stevia. | 200 | 68 | 0.9 | 3 | 5.8 | 2.1 | 0.8 | 28 |
| Beverages | water, coconut cream, pea protein, minerals (tricalcium phosphate), (guar gum, gellan gum, polyphosphates), sunflower lecithin, vitamins (B6, D2, B12), natural aroma and stevia. | 200 | 53 | 0 | 3 | 4.4 | 3.7 | 0 | 50 |
| Beverages | water, almonds, coconut cream, pea protein, minerals (tricalcium phosphate), vitamins B6 and B12, (guar gum, gellan gum), sunflower lecithin, natural aroma and stevia. | 200 | 70 | 1 | 3 | 6 | 2.3 | 1 | 96 |
| Beverages | water, cashews, minerals (tricalcium phosphate), vitamins B6 and B12, (guar gum, gellan gum), sunflower lecithin, natural aroma and stevia. | 200 | 88 | 4 | 3 | 6.6 | 2.9 | 0.8 | 69 |
| Beverages | water, coconut cream, pea protein, minerals (tricalcium phosphate), vitamins B6 and B12, (guar gum, gellan gum), sunflower lecithin, natural aroma and stevia. | 200 | 58 | 0.8 | 3 | 4.8 | 4 | 0 | 119 |
| Beverages | water, oats, sunflower oil , tricalcium phosphate and salt . | 200 | 108 | 13 | 1.1 | 5.8 | 0.6 | 2.3 | 121 |
| Beverages | water, oats, sunflower oil , tricalcium phosphate, dipotassium phosphate, dicalcium phosphate, calcium carbonate and salt . | 200 | 145 | 18 | 1.5 | 7.5 | 0.8 | 3.2 | 121 |
| Beverages | rice , calcium, inulin, sunflower oil, salt, natural aroma, xanthan gum | 200 | 117 | 27 | 0 | 1 | 0.2 | 0 | 36 |
| Beverages | water, rice (15%), sunflower oil , almonds e salt | 200 | 111 | 20 | 0 | 3 | 0.6 | 0 | 30 |
| Beverages | rice , calcium, sunflower oil , salt, natural aroma, xanthan gum. | 200 | 117 | 27 | 1 | 1 | 0.2 | 0.6 | 36 |
| Beverages | water, oats, sunflower oil, calcium and salt | 200 | 145 | 18 | 1.5 | 7.5 | 0.8 | 3.2 | 121 |
| Beverages | rice , calcium, inulin, sunflower oil , salt, natural aroma, xanthan gum | 200 | 117 | 27 | 0 | 1 | 0.2 | 0 | 36 |
| Beverages | rice , oat fiber, oats, calcium, sunflower oil , salt, natural aroma and xanthan gum. | 200 | 134 | 27 | 4.6 | 0.8 | 0.2 | 0.8 | 15 |
| Beverages | water, rice, soluble fiber, sunflower oil, calcium, salt, vitamin D, gellan gum, and soy lecitin | 200 | 72 | 11 | 1.1 | 2.4 | 0.2 | 6.2 | 58 |
| Beverages | water, pea isolated protein, demerara sugar, sunflower oil, vitamins | 200 | 113 | 7.5 | 7 | 6.1 | 0.5 | 0 | 176 |
| Beverages | water, rice (6,5%), soluble fiber (polidextrose), sunflower oil, powdered almonds, calcium (tricalcium phosphate), salt, vitamin D (colecalciferol), gellan gum and soy lecitin. | 200 | 79 | 12 | 1.2 | 2.7 | 0.3 | 6.3 | 58 |
| Beverages | water, rice (6,5%), soluble fiber (polidextrose),powdered coconut, sunflower oil, calcium (tricalcium phosphate), salt, vitamin D (colecalciferol), gellan gum e soy lecitin | 200 | 91 | 15 | 1.1 | 2.8 | 0.4 | 6.2 | 60 |
| Beverages | water, oats , soluble fiber, sunflower oil, calcium, salt, vitamin D, gellan gum, and soy lecitin. | 200 | 84 | 16 | 2 | 1.1 | 0.4 | 7.6 | 46 |
| Beverages | water, oats , demerara sugar, soluble fiber, powdered coconut, calcium, salt, vitamin D, and gellan gum | 260 | 104 | 20 | 3 | 1.3 | 0.4 | 10 | 61 |
| Beverages | rice flour e oat flour, pea protein, polidextrose, demerara sugar, soy lecitin e and dipotassium phosphate. | 25 | 79 | 8.6 | 7.3 | 1.3 | 0.2 | 4.5 | 158 |
| Beverages | rice flour e oat flour, pea protein, polidextrose, demerara sugar, soy lecitin e and dipotassium phosphate. | 25 | 73 | 11 | 5 | 1 | 0 | 5.9 | 104 |
| Beverages | rice flour e oat flour, pea protein, polidextrose, demerara sugar, soy lecitin e and dipotassium phosphate. | 200 | 120 | 15 | 4.8 | 4.6 | 0.4 | 2.7 | 104 |
| Beverages | water, oats, canola oil, bipotassium phosphate, calcium carbonate, salt, vitamin d2, b2 and b12 | 200 | 97 | 8.4 | 2.3 | 6 | 1 | 0 | 18 |
| Beverages | water, oats, salt | 200 | 66 | 10 | 2.7 | 1.5 | 0.4 | 1 | 80 |
| Beverages | water, oats, vanilla extract and salt | 200 | 99 | 16 | 4.1 | 1.9 | 0.5 | 1.1 | 80 |
| Beverages | water, oats, cocoa and salt | 200 | 104 | 18 | 3.6 | 2 | 0.5 | 1 | 80 |
| Beverages | water, oats, calcium carbonate and salt | 200 | 82 | 14 | 3.3 | 1.6 | 0.5 | 0.9 | 80 |
| Beverages | water, oats, canola oil, bipotassium phosphate, calcium carbonate, salt, vitamin d2, b2 and b12 | 200 | 97 | 8.4 | 2.3 | 6 | 1 | 0 | 18 |
| Beverages | almonds , sugar, calcium carbonate, salt , vitamins A, D e E, potassium citrate, gellan gum and tara gum, sunflower lecithin | 200 | 51 | 6.3 | 0.9 | 2.5 | 0 | 0 | 39 |
| Beverages | almonds, powdered coconut, sugar, calcium carbonate, salt , vitamins A, D and E, potassium citrate, gellan gum and tara gum e sunflower lecithin. | 200 | 98 | 17 | 1.3 | 2.8 | 0.3 | 0 | 40 |
| Beverages | almonds, sugar, calcium carbonate, salt , vitamins A, D e E, potassium citrate, gellan gum and tara gum e sunflower lecithin. | 200 | 70 | 11 | 0.9 | 2.5 | 0 | 0 | 39 |
| Beverages | almonds, calcium carbonate, salt , vitamins A, D e E, potassium citrate, tara gum e gellan gum, sunflower lecithin | 200 | 30 | 0.9 | 0.9 | 2.5 | 0 | 0 | 39 |
| Beverages | almonds, sugar, powdered coconut, calcium carbonate, salt, vitamins A, D and E, potassium citrate, gellan gum and tar agum and sunflower lecithin. | 200 | 58 | 8.5 | 0.9 | 2.3 | 0 | 0 | 39 |
| Beverages | almonds , powdered coconut, calcium carbonate, salt , vitamins A, D and E, potassium citrate, tara gum e gellan gum e sunflower lecithin. | 250 | 44 | 1.4 | 1.6 | 3.5 | 0.4 | 0 | 50 |
| Beverages | water, sugar, almonds, calcium carbonate, carob gum e gellan gum and sunflower lecithin. | 200 | 21 | 4.7 | 1 | 2 | 0 | 0 | 122 |
| Beverages | water, sugar, almonds, calcium carbonate, carob gum and gellan gum and sunflower lecithin. | 200 | 48 | 6.8 | 6.8 | 2 | 0 | 0 | 164 |
| Beverages | water, cashews, sugar, calcium carbonate, carob gum and gellan gum, sunflower lecithin | 200 | 59 | 7.5 | 0.9 | 2.8 | 0.5 | 0 | 145 |
| Beverages | water, sugar, almonds, cocoa, calcium carbonate, carob gum and gellan gum, ascorbic acid, sunflower lecithin | 200 | 84 | 15 | 1.2 | 2.1 | 0.3 | 1 | 165 |
| Beverages | water, almonds, sugar,  calcium carbonate, carob gum and gellan gum, sunflower lecithin | 200 | 55 | 4.7 | 0 | 3.7 | 3.3 | 0 | 150 |
| Beverages | water, rice, sunflower oil, calcium carbonate , salt, tricalcium phosphate and sodium citrate. | 200 | 74 | 15 | 0 | 1.55 | 0 | 0 | 40 |
| Beverages | water, rice, sunflower oil, almonds, natural aroma, salt, xanthan gum, tricalcium phosphate e sodium citrate. | 200 | 74 | 15 | 0 | 1.55 | 0 | 0 | 40 |
| Beverages | water, rice, sunflower oil, peanuts, natural cheese aroma, salt , xanthan gum, tricalcium phosphate and sodium citrate. | 200 | 74 | 15 | 0 | 1.55 | 0 | 0 | 40 |
| Beverages | water, rice, sunflower oil, cashews, natural cheese aroma, salt, xanthan gum, tricalcium phosphate and sodium citrate. | 200 | 74 | 15 | 0 | 1.55 | 0 | 0 | 40 |
| Beverages | water, rice, sunflower oil, powdered coconut, salt, xanthan gum, tricalcium phosphate, sodium citrate and stevia | 200 | 74 | 15 | 0 | 1.55 | 0 | 0 | 40 |
| Beverages | water, rice, sunflower oil, grated coconut, water, salt, tricalcium phosphate and sodium citrate. | 200 | 74 | 15 | 0 | 1.55 | 0 | 0 | 40 |
| Beverages | water, rice, sunflower oil, salt , natural cheese aroma, tricalcium phosphate and sodium citrate. | 200 | 74 | 15 | 0 | 1.55 | 0 | 0 | 40 |
| Beverages | rice, polidextrose, calcium carbonate, salt | 30 | 114 | 25 | 2 | 0 | 0 | 1.5 | 45 |
| Beverages | rice, polidextrose, calcium carbonate, salt | 30 | 114 | 25 | 2 | 0 | 0 | 1.5 | 45 |
| Beverages | rice, inulin, banana pulp, calcium carbonate, salt, xanthan gum, stevia | 30 | 114 | 25 | 2 | 0 | 0 | 1.5 | 45 |
| Beverages | rice, powdered coconut, inulin, calcium carbonate, salt, xanthan gum, stevia | 30 | 114 | 25 | 2 | 0 | 0 | 1.5 | 45 |
| Beverages | rice, inulin, strawberries, calcium carbonate, salt, xanthan gum, beet natural coloring and stevia. | 30 | 114 | 25 | 2 | 0 | 0 | 1.5 | 45 |
| Beverages | water, oats, cashews, salt and natural aroma. | 200 | 78 | 8.5 | 2.4 | 3..8 | 0.7 | 0 | 85 |
| Beverages | water and cashews | 200 | 90 | 3 | 3 | 7.3 | 1.4 | 0 | 0 |
| Beverages | water, cashews and coconut | 200 | 80 | 3.1 | 2 | 6.6 | 1.4 | 0 | 0 |
| Beverages | water and almonds | 200 | 57 | 1.7 | 1.9 | 4.7 | 0.4 | 2 | 39 |
| Beverages | water, cashews , demerara sugar and cocoa | 200 | 150 | 18 | 3 | 7.3 | 1.5 | 1.2 | 0 |
| Beverages | water, oat flour, calcium carbonate and salt | 200 | 75 | 16 | 0 | 1.2 | 0 | 0 | 99 |
| Beverages | water, cashews, pea protein, brazil nuts e tricalcium phosphate | 200 | 100 | 2.3 | 7 | 7 | 1.3 | 0.9 | 95 |
| Beverages | water, cashews and brazil nuts | 200 | 80 | 3.3 | 2.1 | 6.5 | 1.4 | 0 | 0 |
| Beverages | water, peanuts and cashews | 200 | 109 | 3.1 | 5.4 | 8.3 | 0.7 | 1.2 | 0 |
| Beverages | water, oat flour, coconut cream, calcium (tricalcium phosphate), gellan gum, guar gum, vitamins (D, E, B12) | 200 | 29 | 0.7 | 0.7 | 1.7 | 1.4 | 0.4 | 0 |
| Beverages | water, almonds, calcium (tricalcium phosphate), gellan gum, guar gum, vitamins (D, E, B12) | 200 | 36 | 1.3 | 1.3 | 3.2 | 0.2 | 0 | 0 |
| Beverages | water, oats, calcium (tricalcium phosphate), natural aroma, gellan gum, guar gum, vitamins (D, E, B12). | 200 | 23 | 4 | 0.8 | 0.4 | 0 | 0.6 | 0 |
| Beverages | water, macadamia nuts, almonds and cashews | 200 | 82 | 2.2 | 1.6 | 7.6 | 1.5 | 1 | 11 |
| Beverages | water, macadamia nuts and coconut | 200 | 106 | 3 | 3 | 11 | 4.5 | 2 | 11 |
| Beverages | water, macadamia nuts and cashews | 200 | 86 | 3 | 3 | 6 | 1.2 | 2 | 11 |
| Beverages | water, demerara sugar, cashews, calcium carbonate, salt , guar gum and natural aroma | 200 | 41 | 5 | 0.8 | 2 | 0.3 | 0 | 117 |
| Beverages | water, demerara sugar, cashews, cocoa, calcium carbonate, salt, guar gum and natural aroma | 200 | 80 | 12 | 1.5 | 2.9 | 0.5 | 1 | 118 |
| Beverages | cashews , calcium (tricalcium phosphate), salt , vitamin A (retinila palmitate), vitamin d2 (ergocalciferol), natural gellan gum and sunflower lecithin. | 200 | 26 | 1 | 0.7 | 2 | 0.4 | 0.6 | 88 |
| Beverages | almonds , calcium (tricalcium phosphate), salt , vitamin A (retinila palmitate), vitamin d2 (ergocalciferol), natural gellan gum and sunflower lecithin. | 200 | 27 | 1 | 0.8 | 2.1 | 0 | 0.6 | 88 |
| Beverages | water, rice, sunflower oil, calcium (tricalcium phosphate), salt , vitamin A (retinila palmitate), vitamin d2 (ergocalciferol), natural gellan gum | 200 | 71 | 11 | 1.1 | 2.7 | 0 | 0.8 | 72 |
| Beverages | water, oats, sunflower oil, calcium (tricalcium phosphate), salt , vitamin A (retinila palmitate), vitamin d2 (ergocalciferol), natural gellan gum | 200 | 99 | 14 | 2.2 | 3.9 | 0.3 | 2.5 | 72 |
| Beverages | water, rice, demerara sugar, powdered coconut, sunflower oil, calcium (tricalcium phosphate), salt , vitamin A (retinila palmitate), vitamin d2 (ergocalciferol), natural gellan gum | 200 | 106 | 19 | 2.2 | 3.7 | 0.3 | 2.2 | 91 |
| Beverages | water, rice, powdered coconut, sunflower oil, calcium (tricalcium phosphate), salt , vitamin A (retinila palmitate), vitamin d2 (ergocalciferol), natural gellan gum. | 200 | 93 | 16 | 1.1 | 2.8 | 0 | 0.8 | 74 |
| Beverages | water, soy extract, sugar, calcium (tricalcium phosphate), sodium chloride (salt), carrageenan gum, carboxymethylcellulose and sodium citrate. | 200 | 95 | 12 | 5.4 | 2.9 | 1.7 | 0.8 | 95 |
| Beverages | water, peanuts and cashews | 200 | 123 | 4.8 | 5.1 | 9.3 | 1.2 | 1.5 | 8.3 |
| Beverages | water and cashews | 200 | 108 | 5.4 | 3.8 | 7.9 | 1.5 | 0 | 9.1 |
| Beverages | water and cashews | 200 | 84 | 3.9 | 2.8 | 6.3 | 1.1 | 0 | 3.1 |
| Yogurts | water, coconut cream, sugar, modified starch, soluble fiber, tricalcium phosphate, xanthan gum, natural aroma, potassium sorbate. | 200 | 142 | 22 | 0.8 | 5.6 | 5.3 | 1.1 | 23 |
| Yogurts | water, coconut cream, sugar, water, strawberries, maltodextrin, modified starch, anatto natural coloring, xanthan gum, potassium sorbate, lactic acid, modified starch, soluble fiber, tricalcium phosphate, xanthan gum, potassium sorbate. | 200 | 149 | 24 | 0.8 | 5.6 | 5.3 | 1.1 | 24 |
| Yogurts | water, coconut cream, sugar, banana, papaya, apple (water, maltodextrin, banana, apple and papaya, modified starch, anatto natural coloring, lactic acid and potassium sorbate), modified starch, soluble fiber, tricalcium phosphate, xanthan gum, potassium sorbate | 200 | 149 | 24 | 0.8 | 5.6 | 5.3 | 1.1 | 24 |
| Yogurts | water, coconut cream, modified starch, soluble fiber, tricalcium phosphate , xanthan gum, natural aroma , potassium sorbate, stevia. | 200 | 92 | 6.4 | 0.9 | 7 | 6.6 | 2.9 | 25 |
| Yogurts | water, coconut cream, strawberries, maltodextrin, modified starch, anatto natural coloring, , xanthan gum, potassium sorbate and lactic acid, modified starch, soluble fiber, tricalcium phosphate, xanthan gum, potassium sorbate, stevia. | 200 | 100 | 8.2 | 1 | 7 | 6.6 | 2.9 | 25 |
| Yogurts | water, coconut cream, sugar, modified starch, pectin, potassium sorbate | 150 | 145 | 20 | 1 | 7 | 6 | 0.5 | 26.4 |
| Yogurts | water, coconut cream, sugar, strawberriess, modified starch, pectin, apple juice, grape juice, beet coloring, potassium sorbate | 150 | 148 | 21 | 1 | 7 | 6 | 0.7 | 26.5 |
| Yogurts | water, coconut cream, pea protein and soy protein, sugar, strawberries (water, sugar, beet natural coloring, strawberries, pectin, citric acid, natural aroma, potassium sorbate), soluble fiber, tricalcium phosphate , sunflower lecithin, natural aroma, potassium sorbate and stevia | 250 | 156 | 11.7 | 14 | 5.9 | 4.7 | 2.6 | 152 |
| Yogurts | water, coconut cream, pea protein, sugar, cocoa, (guar gum, gellan gum, poliphosphates), sunflower lecithin, natural aroma and stevia. | 250 | 123 | 11 | 10 | 5.3 | 4 | 0.4 | 135 |
| Yogurts | water, coconut cream, pea protein and soy protein, sugar, peanuts (water, sugar, natural cheese aroma, peanuts, caramel coloring, pectin, citric acid, potassium sorbate), soluble fiber, tricalcium phosphate , sunflower lecithin, pectin, natural aroma, potassium sorbate, stevia | 250 | 152 | 11 | 14 | 5.4 | 4.2 | 2.6 | 153 |
| Yogurts | almonds, palm oil, starch, carrageenan gum, potassium citrate, tara gum, tripotassium phosphate and sunflower lecithin. | 15 | 27 | 0 | 0 | 3 | 1.3 | 0 | 21 |
| Cheese | water, modified potato starch, vegetal fat, artificial cheese flavoring, anatto coloring, salt, spices, pepper and potassium sorbate. | 50 | 124 | 5.1 | 0 | 11.53 | 1.33 | 0 | 33.83 |
| Cheese | water, modified potato starch, vegetal fat, artificial cheese flavoring, anatto coloring, salt, spices, pepper and potassium sorbate. | 50 | 124 | 5.1 | 0 | 11.53 | 1.33 | 0 | 33.83 |
| Cheese | water, modified potato starch, vegetal fat, artificial cheese flavoring, anatto coloring, salt, spices and pepper, potassium sorbate. | 50 | 124 | 5.1 | 0 | 11.53 | 1.33 | 0 | 33.83 |
| Cheese | water, soy oil, soy extract, modified starch, yeast, salt, potassium sorbate, citric acid, natural coloring, carboxymethylcellulose, calcium disodium, guar gum, xanthan gum, tocopherol and natural cheese aroma | 10 | 31 | 0.6 | 0.3 | 3 | 0.5 | 0 | 34 |
| Cheese | water, soy oil, soy extract and modified starch, yeast, salt, potassium sorbate, citric acid, natural anatto coloring, carboxymethylcellulose, calcium disodium, guar gum, xanthan gum , tocopherol and natural cheese aroma | 10 | 25 | 0.7 | 0.3 | 2.4 | 0.4 | 0 | 44 |
| Cheese | water, soy oil, soy extract, modified starch, yeast, salt, potassium sorbate, citric acid, carboxymethylcellulose , calcium disodium, guar gum e xanthan gum, tocopherol and natural cheese aroma | 10 | 29 | 0.7 | 0.3 | 2.8 | 0.4 | 0 | 12 |
| Cheese | water, soy oil, soy extract e modified starch, yeast, salt, potassium sorbate, citricacid, carboxymethylcellulose, calcium disodium, guar gum, xanthan gum, tocopherol and natural cheese aroma | 10 | 30 | 0.7 | 0.3 | 2.9 | 0.4 | 0 | 42 |
| Cheese | water, peanuts, chickpeas, vinegar, salt | 100 | 178 | 8.7 | 8.4 | 12.2 | 2.2 | 0 | 23.6 |
| Cheese | water, peanuts, chickpeas, vinegar, curry, garlic, salt | 100 | 178 | 8.7 | 8.4 | 12.2 | 2.2 | 0 | 23.6 |
| Cheese | water, rice flour, sunflower oil, coconut oil, nutritional yeast, mustard, lactic acid, salt | 100 | 280 | 23.3 | 3 | 19.7 | 1 | 2.7 | 386 |
| Cheese | water, peanuts, chickpeas, lupin, sunflower oil, wine, cassava starch, coconut oil, seaweed extract, nutritional yeast, vinegar, mustard, salt | 100 | 170 | 6 | 4.7 | 11.3 | 2.7 | 2.7 | 160 |
| Cheese | water, peanuts, chickpeas, lupin, sunflower oil, wine, cassava starch, coconut oil, seaweed extract, nutritional yeast, vinegar, mustard, salt | 100 | 170 | 6 | 4.7 | 11.3 | 2.7 | 2.7 | 160 |
| Cheese | water, cashews, coconut oil , modified starch, salt , vitamins B6 and B12, yeast extract | 30 | 82 | 2.7 | 1.5 | 7.2 | 4.9 | 0 | 72 |
| Cheese | water, cashews, coconut oil , modified starch, salt , fine herbs, pesto sauce, vitamins (B6 and B12) | 30 | 83 | 2.9 | 1.5 | 7.3 | 4.9 | 0 | 77 |
| Cheese | water, cashews, modified potato starch, modified cassava starch, coconut oil , salt , yeast extract, vitamins (B6 and B12), carrageenan gum, lactic acid potassium sorbate and anatto natural coloring. | 30 | 95 | 9.1 | 2 | 5.7 | 0.8 | 0.4 | 15.4 |
| Cheese | water, cashews, potato starch, coconut oil , salt , yeast extract, vitamins B6 and B12, lactic acid, potassium sorbate and anatto natural coloring. | 30 | 89 | 8.2 | 1.9 | 5.4 | 0.8 | 0.4 | 15.4 |
| Cheese | water, cashews, modified potato starch, modified cassava starch, coconut oil , salt , yeast extract, vitamins B6 and B12, carrageenan gum, lactic acid, potassium sorbate and anatto natural coloring. | 30 | 93 | 8.8 | 1.9 | 5.5 | 0.8 | 0.4 | 15.4 |
| Cheese | water, cashews, coconut oil , modified starch, salt , vitamins B6 and B12, yeastextract, potassium sorbate | 30 | 83 | 2.3 | 1.6 | 7.5 | 5.1 | 0 | 70 |
| Cheese | water, cashews, coconut oil , modified starch, salt , vitamins B6 and B12, fine herbs, potassium sorbate | 30 | 83 | 2.3 | 1.6 | 7.5 | 5.1 | 0 | 71 |
| Cheese | water, cashews, coconut oil , modified starch, salt , vitamins B6 and B12, natural aroma, anatto natural coloring, potassium sorbate | 30 | 83 | 2.3 | 1.6 | 7.5 | 5.1 | 0 | 68 |
| Cheese | cashews, water, probiotics | 100 | 581 | 30.2 | 18.2 | 43.8 | 7.7 | 3.3 | 731 |
| Cheese | cashews, water, coconut oil, nutritional yeast, seaweed extract , cassava starch, probiotics and salt. | 30 | 59 | 2 | 0.9 | 5.6 | 3.8 | 0.2 | 73 |
| Cheese | cashews, water, coconut oil, nutritional yeast, liquid smoke, seaweed extract , cassava starch, probiotics and salt. | 30 | 43 | 1.2 | 0.8 | 4.1 | 2.8 | 0.2 | 48 |
| Cheese | cashews, water , coconut oil, probiotics and salt. | 30 | 100 | 5.4 | 2.9 | 8.5 | 2.3 | 0.5 | 50 |
| Cheese | cashews, water, coconut oil, nutritional yeast, seaweed extract , cassava starch, probiotics, salt and natural anatto coloring. | 30 | 43 | 1.2 | 0.8 | 4.1 | 2.8 | 0.2 | 48 |
| Cheese | cashews, water , coconut oil, nutritional yeast, probiotics and salt. | 100 | 394 | 23 | 13 | 31 | 5 | 3 | 288 |
| Cheese | water, cashews, coconut oil, cassava starch, maltodextrin, salt, seaweed extract and potassium sorbate. | 12 | 30 | 3 | 0.7 | 1.9 | 0.4 | 0.2 | 123 |
| Cheese | cashews, water, lime, coconut oil, onions and garlic, cassava starch, salt, spirulina | 20 | 62.8 | 3.6 | 2 | 4.9 | 1 | 0.4 | 75.2 |
| Cheese | cashews, red bell pepper, lime, water, salt, turmeric | 20 | 57.1 | 3.4 | 1.9 | 4.4 | 0.8 | 0.5 | 54.5 |
| Cheese | cashews, water, lime, salt, garlic, cassava starch, coconut oil, olive oil | 20 | 68.4 | 3.8 | 2.1 | 5.4 | 1.1 | 0.4 | 66.1 |
| Cheese | cashews, salt, coconut oil, probiotics, penicillum candidum | 20 | 62.8 | 3.6 | 2 | 49 | 1 | 0.4 | 75 |
| Cheese | water, potato starch, palm oil, carrot, apple, pumpkin, salt and mozarela aroma. | 30 | 89 | 6.4 | 0 | 7 | 3.1 | 0 | 79 |
| Cheese | cashews, water , coconut oil, seaweed extract , cassava starch, probiotics and salt | 30 | 72 | 2.9 | 1 | 6.4 | 1.3 | 0 | 105 |
| Cheese | cashews, water , coconut oil, seaweed extract , cassava starch, probiotics and salt | 30 | 58 | 3.5 | 1 | 1.3 | 0 | 0 | 205 |
| Cheese | cashews, water , coconut oil, seaweed extract , cassava starch, probiotics and salt. | 30 | 71 | 2.8 | 0.9 | 6.4 | 8.8 | 0 | 63 |
| Cheese | cashews, water , coconut oil, seaweed extract , cassava starch, probiotics and salt | 30 | 78 | 3.4 | 1 | 7.2 | 5.3 | 0 | 99 |
| Cheese | cashews, water , coconut oil, seaweed extract , cassava starch, probiotics, spices and salt | 30 | 76 | 3.4 | 0.9 | 6.8 | 5 | 0 | 156 |
| Cheese | water, cashews, coconut oil , starch, lime juice, salt xanthan gum and potassium sorbate | 15 | 36 | 1 | 0.3 | 3 | 0.5 | 0.25 | 110 |
| Cheese | water, cashews, sunflower oil, starch, lime juice, salt , xanthan gum, nutritional yeast and potassium sorbate | 10 | 38 | 0.8 | 0.3 | 3.8 | 0.5 | 0 | 44 |
| Cheese | cashews, potato starch, coconut oil , salt, xanthan gum, natural anatto coloring and potassium sorbate. | 15 | 51 | 3.8 | 0.7 | 3.9 | 0.1 | 0 | 185 |
| Cheese | water, cashews, sunflower oil, seaweede xtract , salt , and lactic acid | 15 | 36 | 1.2 | 0.4 | 3.3 | 0.2 | 0.5 | 82 |
| Cheese | water, cashews, sunflower oil, potato starch, coconut oil , cassava starch, salt , yeast, natural anatto coloring, carrageenan gum, lactic acid, tricalcium phosphate and xanthan gum | 15 | 47 | 2.8 | 0.1 | 3.9 | 0.4 | 0.1 | 93 |
| Cheese | water, cashews, sunflower oil, potato starch, coconut oil , cassava starch, salt , yeast, carrageenan gum, lactic acid, tricalcium phosphate, xanthan gum and natural anatto coloring. | 15 | 47 | 2.8 | 0.1 | 3.9 | 0.4 | 0.1 | 93 |
| Cheese | water, cashews, sunflower oil, potato starch, coconut oil , cassava starch, salt , carrageenan gum, lactic acid tricalcium phosphate, xanthan gum, natural cheese aroma and natural anatto coloring | 15 | 49 | 3.1 | 0.1 | 3.8 | 0.4 | 0.1 | 97 |
| Cheese | cashews, water, spices, probiotics | 100 | 581 | 30.2 | 18.2 | 43.8 | 7.7 | 3.3 | 731 |
| Mayonnaise | water, vegetable oil, sugar, modified starch, salt, vinegar, soy extract, potassium sorbate, guar gum and xanthan gum, lactic acid e phosphoric acid, calcium disodium | 12 | 28 | 1.4 | 2.5 | 0.4 | 0 | 0 | 125 |
| Mayonnaise | soy oil, modified starch, sugar, vinegar, salt, lactic acid, xanthan gum, potassium sorbate, calcium disodium | 12 | 20 | 0 | 0 | 1.8 | 0.3 | 0 | 94 |
| Mayonnaise | vegetableoil, water, guar gum, aquafaba, cornstarch, vinegar, demerara sugar, salt, spices (mustard and garlic), lime juice, xanthan gum, lactic acid, and calcium disodium | 12 | 70 | 0 | 0 | 7.6 | 1.3 | 0 | 61 |
| Mayonnaise | vegetable oil, water, aquafaba, vinegar, spices (garlic, pepper e mustard), demerara sugar, salt, mustard, cornstarch, lime juice, lactic acid, and calcium disodium | 12 | 71 | 0.6 | 0 | 7.6 | 1.3 | 0 | 62 |
| Mayonnaise | vegetable oil, water, black olives, water, guar gum, aquafaba, corn starch, vinegar, demerara sugar, salt, spices (mustard and garlic), lime juice, xanthan gum, lactic acid, and calcium disodium. | 12 | 57 | 0.6 | 0 | 6.1 | 1 | 0 | 74 |
| Mayonnaise | vegetable oil, water, aquafaba, corn starch, demerara sugar, vinegar, salt, spices (mustard, garlic, red pepper e white pepper), lime juice, lactic acid, xanthan gum, and calcium disodium. | 12 | 56 | 0.6 | 0 | 6 | 1 | 0 | 71 |
| Mayonnaise | water, canola oil, vinegar, starch, pea protein, sugar, salt, mustard, pepper, lactic acid, lime juice, natural aroma, garlic, onion, potassium sorbate and disodium calcium. | 12 | 33 | 0.6 | 0.4 | 3.3 | 0.3 | 0 | 74 |
| Mayonnaise | water, vegetable oil, modified starch, vinegar, sugar, salt, potassium sorbate, lime juice, xanthan gum, calcium disodium and citric acid | 12 | 25 | 0 | 0 | 3.9 | 0.6 | 0 | 89 |
| Mayonnaise | soy oil, modified starch, sugar, vinegar, salt, isolated vegetable protein, hydrolyzed vegetable protein, xanthan gum, citric acid, lactic acid, potassium sorbate, onion, garlic, lime juice, calcium disodium | 12 | 21 | 0 | 0 | 1.8 | 0 | 94 | 0 |
| Mayonnaise | water, soy, sunflower oil, corn starch, salt, vinegar, potassium sorbate | 40 | 84 | 5.2 | 0.5 | 5.1 | 0.6 | 0 | 194 |
| Mayonnaise | water, canola oil, vinegar, starch, pea protein, sugar, salt, mustard, lactic acid, lime juice, natural aroma, garlic, onion, potassium sorbate and disodium calcium. | 12 | 33 | 0.6 | 0.4 | 3.3 | 0.3 | 0 | 74 |
| Mayonnaise | water, canola oil, vinegar, starch, pea protein, sugar, salt, mustard, natural aroma, lactic acid, lime juice, natural aroma, garlic, onion, potassium sorbate and disodium calcium. | 12 | 33 | 0.6 | 0.4 | 3.3 | 0.3 | 0 | 74 |
| Mayonnaise | water, canola oil, vinegar, avocado, starch, pea protein, sugar, salt, mustard, lactic acid, lime juice, natural aroma, garlic, onion, potassium sorbate and disodium calcium. | 12 | 33 | 0.6 | 0.4 | 3.3 | 0.3 | 0 | 74 |
| Mayonnaise | water, canola oil, vinegar, starch, pea protein, sugar, salt, mustard, lactic acid, lime juice, natural aroma, garlic, onion, potassium sorbate and disodium calcium. | 12 | 33 | 0.6 | 0.4 | 3.3 | 0.3 | 0 | 74 |
| Eggs | pea starch, pea protein and sodium bicarbonate | 10 | 25 | 3.3 | 4.1 | 0.8 | 0 | 1.4 | 48 |
| Eggs | isolated rice protein, chick peas and golden flaxseed | 7 | 28 | 1.2 | 3.1 | 1.2 | 0 | 0.9 | 0 |
